# Anti-vascular Endothelial Growth Factor Outpatient Treatment Patterns in Patients with Exudative Age-related Macular Degeneration from a Japanese Hospital Claims Database

**DOI:** 10.36469/9887

**Published:** 2014-07-08

**Authors:** Tomohiro Iida, Aya Narimatsu, Kenji Adachi, Edward CY Wang

**Affiliations:** 1 Department of Ophthalmology, Tokyo Women’s Medical University, Tokyo, Japan; 2 Economics and Outcomes Research, Bayer Yakuhin Ltd., Osaka, Japan

**Keywords:** vegf, real-world, prn, treatment pattern, anti-vegf, age-related macular degeneration

## Abstract

**Purpose:** To identify outpatient treatment patterns of patients with exudative age-related macular degeneration (AMD) who received approved anti–vascular endothelial growth factor (VEGF) therapy, using real-world data from hospitals in Japan.

**Methods:** A hospital claims database was retrospectively reviewed for patients diagnosed with exudative AMD who were treated with anti-VEGF therapy in the outpatient setting between January 2010 and December 2012. Within a treatment period of at least 12 months, the frequency of anti-VEGF injections and AMD-related visits, and time intervals between AMD-related visits and anti-VEGF injections were captured for patients who had neither cataracts nor glaucoma. “Loading dose regimen” was defined as the first 2 or 3 monthly doses and “PRN maintenance regimen” (where PRN=pro re nata) was defined as the entire period of time after the loading doses had been administered.

**Results:** Claims data were collected from a total of 219 patients from 13 of 126 hospitals: 217 treated with ranibizumab (8 received pegaptanib as well), 2 with aflibercept. Of these, 68 patients were treated for at least 12 months (all with ranibizumab PRN), and 29 had neither cataracts nor glaucoma and were included in the treatment pattern analysis. These 29 patients received a mean of 3.8 injections in the first 12 months and another 2.5 injections in the second 12 months of treatment. The average number of all outpatient visits was 16.1 in the first 12 months and 13.7 in the second 12 months, and an average of 11.6 days elapsed between injections and the previous outpatient monitoring visits using a PRN schedule.

**Conclusion:** In a real-world setting in Japan, anti-VEGF PRN injections are administered less frequently than in clinical trials, and with time between monitoring and re-injection visits. Nonetheless, patients still visit the hospital frequently, which can significantly burden patients, caregivers, and healthcare providers.

## INTRODUCTION

In Japan, 1.64 million adults were estimated to be visually impaired in 2007, and this figure is projected to increase to almost 2 million people by 2050.[1] About 11% of the cases of visual impairment are due to age-related macular degeneration (AMD).[1] Epidemiological data from a representative population in Japan showed that exudative AMD, which is the form of AMD responsible for most of the severe vision loss caused by AMD, is more common than dry AMD in Japan.[2,3] Loss of central vision from exudative AMD impairs patients' ability to read, drive, identify faces, watch television, navigate stairs safely, and complete many other daily tasks we typically take for granted (eg, grocery shopping, preparing meals).[4,5] These impairments can result in a devastating loss of independence, decreased quality of life (QoL), and depression.[6-9]

Anti–vascular endothelial growth factor (VEGF) agents have been found to increase visual acuity in patients with exudative AMD.[10-14] In Japan, three agents are approved for treatment: pegaptanib (Macugen®; Pfizer Japan Inc., Tokyo, Japan), ranibizumab (Lucentis®; Novartis Pharma K.K., Tokyo, Japan), and aflibercept (Eylea®; Bayer Yakuhin, Ltd., Osaka, Japan). Now that multiple anti-VEGF options are available, optimizing dosing regimens is important to alleviate treatment burden.

Approved in 2009, ranibizumab is meant to be administered as a 0.5-mg monthly injection for 3 months (the loading dose), with subsequent injections administered on a pro re nata (PRN) basis based on clinical symptoms.[15] With a PRN dosing regimen, monthly visits are still necessary to monitor symptoms and assess when further injections may be needed,[16-20] which, while potentially reducing the number of injections, does not help ease the patients' and caregivers' office visit burdens.[5,21] More recently, at the end of 2012, aflibercept was approved in Japan to be administered as a 2-mg monthly injection for 3 months (the loading dose), followed by regular bimonthly injections.[22]

It is unknown whether actual dosing intervals used in real-world clinical practice are lower than those reported in clinical trials, and whether they may be lower than would be ideal to maintain visual acuity. This retrospective, observational study aimed to identify outpatient treatment patterns in patients with exudative AMD who were treated with anti-VEGF agents in Japanese hospitals.

## PATIENTS AND METHODS

This retrospective, observational study was conducted using a hospital claims database stored in hospital electronic information systems constructed by Medical Data Vision Co., Ltd. (MDV; Tokyo, Japan). The MDV database covers approximately 4.84 million patients in 126 hospitals in Japan, with the number of beds ranging from 20 to more than 1000 located in several regions of the country, and including about 8% of all the acute phase hospitals, except university hospitals, using the Diagnostic Procedure Combination (DPC) payment system / Per-Diem Payment System (PDPS) in Japan.[23] (internal communication, Medical Data Vision Co., Ltd, 2013) The MDV database contains an anonymized patient identifier, as well as patient gender, birth year, department visited, date of medical service, diagnosis codes, hospitalization, medical procedures and test orders, operations, and prescriptions.

The study included patients initially diagnosed with AMD in hospitals between January 1, 2010 and December 31, 2012. AMD was identified by Japanese disease code "8831472" for each patient. This disease code is linked to ICD 10, H35.3. Although AMD has two classifications, exudative AMD and dry AMD, no patients were coded with either of them. Thus, to supplement the information provided by disease codes, anti-VEGF drug prescription data were used to define patients with exudative AMD. Patients received any anti-VEGF agent

(namely, ranibizumab, pegaptanib, or aflibercept). As bevacizumab is not approved for exudative AMD treatment in Japan, no information on bevacizumab use was available in the database.

Patients were excluded if they were aged less than 50 years, if they were treated at an ophthalmology department in the same hospital before the initial diagnosis, or if they received any of the anti-VEGF agents as inpatient therapy. In this way, the study aimed to articulate an accurate frequency of outpatient visits related to anti-VEGF treatment patterns. In addition, as the treatment of cataracts and glaucoma have the potential to lead to outpatient ophthalmology visits that are unrelated to treatments for exudative AMD, patients with either of these comorbidities were considered ineligible for the treatment pattern analysis to avoid confounding effects of any treatments other than the ones of interest for exudative AMD or additional office visits for other reasons.

To identify treatment patterns in patients with exudative AMD with approved anti-VEGF therapy, the following outcomes were assessed: 1) frequency of anti-VEGF injections received; 2) frequency of AMD-related visits; 3) distribution of the time interval between AMD-related visits with and without anti-VEGF injections; and 4) distribution of the time interval of anti-VEGF injections.

In Japan, ranibizumab is indicated to be administered using a loading dose consisting of 3 monthly intravitreal injections, followed by a PRN regimen that is adjusted depending on symptoms.[15] Therefore, for the purposes of the treatment pattern analysis, "loading dose regimen" is defined as the first 2 or 3 monthly doses, "beginning of PRN maintenance regimen" is defined as the first dose after the loading dose injections have been administered, and "PRN maintenance regimen" is defined as the entire period of time after the loading doses have been administered (Figure 1). Patients who received only 1 monthly injection after initial diagnosis were considered to not have completed the loading dose regimen. In order to investigate anti-VEGF treatment patterns for exudative AMD, a treatment period of 12 months was chosen as the minimum duration required to encompass the loading dose and PRN maintenance regimens. The treatment period was defined as the period of time starting with the first anti-VEGF injection through the last outpatient visit observed for each patient. In addition, "monthly consecutive" dosing within the loading dose regimen was defined as a second injection that was administered within 6 (4+2) weeks after the first injection, and a third injection administered within 6 (4+2) weeks after the second injection. In order to allow for inclusion of patients whose consecutive injections were administered after slightly more time than monthly, perhaps due to difficulties in appointment schedule arrangement, "monthly" was defined as 4 weeks plus an additional 2 weeks.

**Figure 1.** Definitions of the Loading Dose Regimen and the PRN Maintenance Regimen

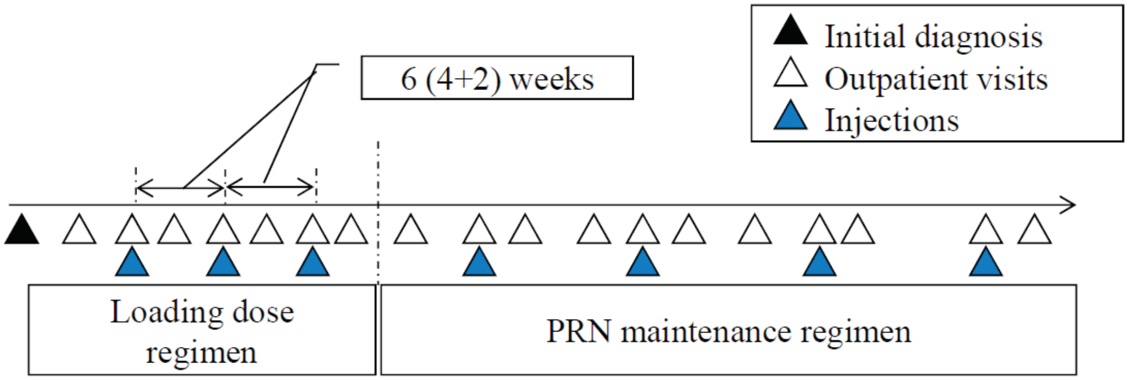

PRN: pro re nata

Statistical analysis was exploratory in nature and used descriptive statistics. Mean and standard deviation (SD) were reported for continuous variables such as age distribution, time intervals, number of visits, and number of injections. The data were stratified by subgroups where appropriate and informative.

## RESULTS

### Patients

Out of the 126 hospitals in the database, claims data from a total of 219 patients were collected from 13 hospitals (Table 1), including 217 patients who received ranibizumab (8 of whom received both ranibizumab and pegaptanib), and 2 patients who received aflibercept. Among the 217 patients who received outpatient treatment (all with ranibizumab), 68 patients were treated for 12 months or more. The main treatment pattern analysis included the 29 patients who were treated for 12 months or more and had neither cataracts nor glaucoma (Figure 2). Eleven of the 29 patients were treated for 24 months or more (Table 2). The 29 patients were treated at 7 hospitals out of the 13 (Table 1). The two patients who received aflibercept did not meet the 12-month treatment inclusion criterion; therefore, detailed analyses of the treatment patterns were completed for the ranibizumab population only.

**Table 1.** Description of 13 Hospitals visited by Study Participants

| | Number of Hospitals | |
|---|---|---|
| **Type of Hospital** | **Overall Population (N=219)** | **Main Treatment Pattern Analysis Population (n=29)** |
| Local Government | 8 | 5 |
| Private Sector | 4 | 2 |
| University | 0 | 0 |
| Other (social insurance association, etc) | 1 | 0 |
| **Number of Patients who visited each Hospital's Department of Ophthalmology in 2012** | | |
| >10,000 Patients | 1 | 1 |
| 5000-9999 Patients | 3 | 2 |
| <5000 Patients | 9 | 4 |
| **Location of Hospital (ordinance designation)** | | |
| Ordinance-Designated Cities | 2 | 1 |
| Other Cities | 11 | 6 |

More of the patients were men, whose mean age was 72.2±8.3 years (median, 75.5; range, 52.0-81.0) (Table 2). The mean age of the women was 74.5±9.2 years (median, 71.0; range, 63.0-90.0), with the majority of the patients being at least 70 years of age. This sex distribution was consistent with that of the long-term epidemiological study completed in Hisayama, Japan, which is representative of the Japanese population as a whole, and found that exudative AMD was more common in men than in women (1.2% vs 0.34%; P<0.01). However, unlike the current study, in which 72.2% of men and 63.6% of women were at least 70 years of age, only 32% of the Hisayama population with exudative AMD was aged 70 years or more, which the authors attributed to the low number of people with late AMD changes in the Hisayama study.[2,3]

**Figure 2.** Patient Disposition

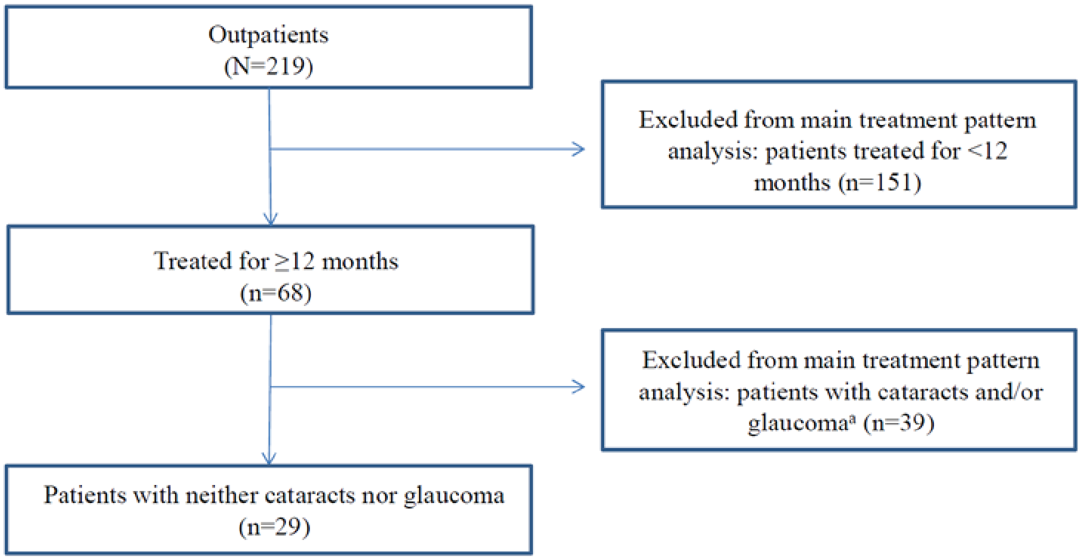

The main focus of the treatment pattern analysis included outpatient data from patients with AMD treated for at least 12 months that would not be confounded by other eye-related comorbidities (n=29). [a]Two patients had both cataract and glaucoma.

**Table 2.** Demographics

| **Patient Characteristics (n=29)** | |
|---|---|
| **Observation period** | **n (%)** |
| ≥12 months | 29 (100) |
| ≥24 months | 11 (38) |
| **Gender** | **n (%)** |
| Men | 18 (62.1) |
| Women | 11 (37.9) |
| **Age (years)** | **Mean±SD** |
| Men | 72.2±8.3 |
| Women | 74.5±9.2 |
| **≥70 years of age** | **n (%)** |
| Men | 13 (72.2) |
| Women | 7 (63.6) |

Data are presented as mean±SD or numbers with percent in parentheses; SD: standard deviation

### Treatment Patterns of Anti-VEGF Agents

In the population of 29 patients who were treated with ranibizumab for 12 months or more and had neither cataracts nor glaucoma, patients received an average of 3.8 injections in the first 12 months and another 2.5 injections in the second 12 months of treatment, for a total of 6.3 in the entire 24-month study period (Table 3). An average of 10.7±6.5 days elapsed between the last outpatient visit before receiving the first PRN maintenance injection, and the mean time interval between injections and the previous outpatient visits in the PRN maintenance regimen period was 11.6±6.6 days. Twenty-five percent of visits occurred after more than a 2-week (14-day) interval, and 10% occurred after more than a 3-week (21-day) interval. Despite the absence of other major eye-related comorbidities, 62.1% of these 29 patients visited the hospital 12 or more times in 12 months for exudative AMD treatment.

The average number of all outpatient visits for these 29 patients was 16.1 in the first 12 months of treatment and 13.7 in the second 12 months. Many injections (51.9%) were followed by next-day office visits. In addition, of the 29 patients in the main treatment pattern analysis, one patient received a single injection of pegaptanib within the study period after the loading dose period (during the PRN maintenance regimen).

**Table 3.** Treatment Pattern Analysis

| Characteristic (n=29) | Mean±SD | Median |
|---|---|---|
| **Average number of injections per year** | | |
| Months 1-12 (n=29) | 3.8±2.8 | 3.0 |
| Months 13-24 (n=11) | 2.5±3.1 | 1.0 |
| **Average number of all outpatient visits** | | |
| Months 1-12 (n=29) | 16.1±9.9 | 14.0 |
| Months 13-24 (n=11) | 13.7±10.1 | 9.0 |
| **Before treatment (n=29)** | | |
| Time interval between first diagnosis and first injection, days | 74.8±115.3 | 28.0 |
| Number of outpatient visits between first diagnosis and first injection | 2.6±3.6 | 1.0 |
| **Loading dose regimen (n=29)** | | |
| Time interval between injections and the previous outpatient visits during loading dose regimen, days | 12.3±6.9 | 13.0 |
| Time interval between injections and the following outpatient visits during loading dose regimen, days | 9.4±11.2 | 1.0 |
| **Beginning of PRN maintenance regimen (n=29)** | | |
| Time interval between the last loading dose injection and the first PRN injection, days | 102.0±116.1 | 63.0 |
| Number of outpatient visits between the last loading dose injection and the first PRN injection | 3.0±2.1 | 3.5 |
| Time interval between the last visit after the last loading dose injection and the first PRN injection, days | 10.7±6.5 | 8.5 |
| **PRN maintenance regimen (n=29)** | | |
| Time interval between injections and the previous outpatient visits during PRN maintenance regimen, days | 11.6±6.6 | 9.0 |
| Time interval between injections and the following outpatient visits during PRN maintenance regimen, days | 18.5±39.5 | 21.0 |
| **Fluorescein angiography (FA) or indocyanine angiography (IA), (n=29)** | | **n (%)** |
| Patients who received FA or IA before the first injection | | 25 (86.2) |

"Loading dose regimen" is defined as the first 2 or 3 monthly doses, "Beginning of PRN maintenance regimen" is defined as the first dose after the loading dose injections have been administered, and "PRN maintenance regimen" is defined as the entire period of time after the loading doses have been administered. Data are presented as mean±SD, medians, or numbers with percent in parentheses.

FA: fluorescein angiography; IA: indocyanine angiography; PRN: pro re nata (as needed); SD: standard deviation

### Treatment patterns of anti-VEGF agents and photodynamic therapy

Out of the 219 patients who received anti-VEGF therapy, 15 patients received photodynamic therapy (PDT) during the study period. Among those 15 patients, PDT was administered with an average of 1.3 PDT administrations per patient. All of these 15 patients received ranibizumab and no one received either pegaptanib or aflibercept, and all PDT was administered as inpatient treatment. Three patients had PDT before the first ranibizumab injection, and 12 had PDT after the loading dose (during the PRN maintenance regimen). Among the three patients who received PDT before their ranibizumab treatment, the average time interval between PDT and the following ranibizumab injection was 146±95 days. Among the 12 patients who received PDT during the patient's PRN maintenance regimen of ranibizumab (after loading dose), the average time interval between the last ranibizumab injection and the following PDT was 38±51 days.

Of the 29 patients in the main treatment pattern analysis, two patients received PDT during the study period. Among those 2 patients, PDT was administered three times each. All PDT was administered as inpatient treatment. One patient had PDT before the first ranibizumab injection, and the other had PDT after the loading dose (while receiving ranibizumab via the PRN maintenance regimen).

## DISCUSSION

This study is the first to report the actual usage of anti-VEGF agents in real-world clinical practice in Japan using a comprehensive medical claims database. In this study, on average, patients received 3.8 injections per year in the first 12 months of treatment, which, for patients who received all three monthly consecutive loading dose injections, equates to three initial monthly doses (the loading dose), plus 0.8 additional injection given within the remaining 9 months via the PRN schedule. Patients later received an average of 2.5 injections in the second 12 months of treatment, with a total average of 6.3 injections administered over the entire study period. Our results show that the real-world number of ranibizumab injections administered is lower than the injection rate reported in clinical trials.

The CATT trial, which included a treatment arm for the ranibizumab PRN dosing scheme, resulted in a mean of seven injections in the first 12 months of treatment, which is almost twice as many injections as found in this study.[24] Similarly, in the HARBOR trial, the mean numbers of injections in the first 12 months were 7.7 for the 0.5-mg group treated with ranibizumab PRN.[25] As was the case with the results reported from the first 12 months, in HARBOR, the mean numbers of injections in the second 12 months for the PRN groups were 5.6 (0.5 mg) and 4.3 (2.0 mg), which were higher than the 2.5 injections seen in our study in the second 12 months alone.[26] The mean number of injections received for the CATT ranibizumab PRN group for the full 24-month treatment period was 12.6, which is exactly twice the number of injections reported in the 24-month treatment period of the current study.[27] Other prospective, interventional trials also reported injection totals that were higher than the ones found in the current study.[16-18,28]

The same phenomenon can be seen with observational data from other trials conducted outside Japan.[29,30] In Japan, on the other hand, two studies conducted at hospitals where retina specialists work revealed that patients received 6.0 injections and 5.2 injections, respectively, in the first 12 months,[19,31] which are higher than the number of injections observed in our study. The results from these two single-center studies and from the current study, which represents data collected from seven different hospitals, albeit with a small absolute number of patients, could suggest that the number of injections given varies across hospitals in Japan.

Trials such as CATT and HARBOR revealed that visual acuity outcomes tend to be slightly lower when patients are treated with a less regular regimen (ie, PRN vs monthly), but patients in the PRN groups of these protocol-driven clinical trials received more injections than our real-world population did.[24,25,27] Studies conducted in observational settings have also shown that the number of injections patients received was strongly related to visual acuity levels. A retrospective chart analysis in Paris, France, found that patients who had a mean of 3.79 injections in 12 months saw a gain in visual acuity of +0.7 letters on the Early Treatment Diabetic Retinopathy Study (ETDRS) scale, which represented more of a stabilization than an improvement.[32,33] An Italian retrospective chart review showed that patients received 4.8 injections in 12 months and experienced visual acuity loss of two letters on the ETDRS scale.[34] In addition, a large, multicenter, retrospective study conducted in Germany showed that the low mean number of injections (4.34) led to modest improvements in visual acuity that were not able to be sustained until the last follow-up visit at 12 months.[35]

In another retrospective study from Cleveland, Ohio, where patients received a mean of 5.2 injections in 12 months, it was discovered that patients who received more frequent injections had higher visual gains than patients with less frequent injections did ($P=0.012$).[36] Recently, a study was conducted to compare the visual acuity outcomes of ranibizumab treatment from an observational cohort with those of the MARINA study cohort.[11,30] Using data from the prospective, observational Fight Retinal Blindness database, the authors showed that an average of 7.3 ranibizumab injections in 12 months appeared to be needed to improve visual acuity to levels similar to those seen in the MARINA study. The authors also noted that 7.3 injections are similar to the number of injections received by the patients in the PRN arm of the CATT trial.[27,30]

In addition to the number of injections, the timing of injections in a PRN dosing scheme may impact outcomes. It is important to note that, in CATT, PRN injections were administered on the same day of the monthly follow-up visits when injections were deemed necessary, whereas our study revealed that 11.6 days elapsed between injections and the previous outpatient visits during the PRN maintenance regimen period, with 25% of visits occurring after more than 2 weeks, and 10% of visits after more than 3 weeks. Likewise, another Japanese treatment pattern study conducted at two hospitals found that injections of ranibizumab were administered an average of 47 and 7 days, respectively, after the injections were first indicated.[37] A main reason for this time interval may be difficulty in booking for intravitreal injections, as some hospitals in Japan require injections to be administered in operating rooms, which may take time to become available. A low injection frequency alone, without time between the indication for treatment and injection administration, can affect outcomes, as was shown from trials such as CATT. Our results indicate that in reality, a patient may not be able to receive necessary injections as quickly as in a protocol-driven clinical trial. It remains to be studied whether this time interval may further compound visual acuity deficits seen in patients with lower injection frequencies.

Infrequent dosing may also negatively impact choroidal neovascular activity and QoL outcomes. An analysis of literature, ongoing clinical trials, and clinical assessments regarding treatment with anti-VEGF agents in 2007 found that less than monthly treatment with the agents available then sometimes led to multiple recurrences of choroidal neovascular activity, which could potentially result in progressive damage of the neurosensory retina and long-term visual impairments.[38] This may help explain why the prospective, observational HELIOS trial showed that real-world PRN ranibizumab treatment in Belgium resulted in lower patient-reported vision-related QoL scores than those found in protocol-driven clinical trials.[11,12,29] As our study showed even lower injection numbers than those seen in HELIOS, vision-related QoL may be affected in this real-world population.

Despite the absence of other major eye-related comorbidities, the 29 patients in the current study visited the hospital 12 or more times in 12 months for exudative AMD treatment with an average of 16.1 visits, and, in seven cases (24.1%), visited the hospital 20 or more times in the first 12 months. The average number of all outpatient visits for these patients was nearly 30 in 24 months, which is more than monthly. In addition, we found in our study that many injections (51.9%) were followed by a visit on the very next day. Therefore, patients who received approximately 4 injections followed those injections with 4 next-day follow-up visits, which, added to 8 more monthly monitoring visits for non-injection months, equals 16 visits in 12 months. According to the Japanese prescribing information, visual acuity should be assessed monthly when using ranibizumab via a PRN maintenance regimen to determine if further injections are needed.15 In reality, as shown by the current study, patients treated in a hospital in Japan are visiting the hospital more frequently than is indicated by Japanese retreatment guidelines and anti-VEGF agent prescribing information.[15,20] Outside of Japan, an observational analysis showed that patients made 9.5 visits in 12 months. As the average injection rate from that study was 7.3, there were only 2.2 injection-free visits.[30] This may represent a significant burden for patients and caregivers in Japan, especially as patients have cited travel to and from office visits as a major barrier to their treatment adherence.[39] In addition, due to the increasing number of patients requiring anti-VEGF treatment in Japan because of an aging population and expanded indications of anti-VEGF agents,[40] a high frequency of visits may limit the number of patients that each healthcare provider is able to treat.

Our real-world study also showed that some patients received concomitant pegaptanib therapy or PDT, although this represented only a few patients of the 29 in the treatment pattern analysis (one who received pegaptanib, two who received PDT). Of the patients who received these concomitant treatments, one injection of pegaptanib was administered during the study period, and the mean number of PDT administrations was three per patient, representing potential additional office visits for these patients, increasing their treatment burden above that seen with study medication administration alone in prospective, interventional clinical trial protocols.

The study has several limitations. Right censoring (ie, determining the set follow-up time ending in December 2012) placed in this study may distort the selection and generalizability of the study cohort. Also, since the date of approval of aflibercept in Japan was so close to the end of the studied diagnosis time periods, we were not able to capture the treatment pattern of this drug. It would be an interesting future research topic to investigate how anti-VEGF treatment patterns evolve over time after the introduction of new therapy options. Few patients in the total outpatient population had a treatment period of sufficient duration (at least 12 months) to include in the treatment pattern analysis, because some of the hospitals started providing their data to the MDV database only recently. In addition, in this study, the total number of patients evaluated was small in size, hence not allowing for a representative distribution of the study population, limiting conclusions that can be drawn. Finally, it is unknown whether each patient was treating one eye or two for exudative AMD, which could affect the number of injections and outpatient visits.

In conclusion, this study showed from real-world data in Japan that anti-VEGF injections, when given using a PRN schedule, are administered less frequently than in clinical trials. The time interval between the indication for treatment and the administration of the injection observed in this study has the potential to lead to progressive visual deficits. Additionally, despite low injection rates, the patients visited the hospital frequently, which can be a significant burden to patients and caregivers. A regular dosing schedule with fewer hospital visits may help reduce the burden to patients, caregivers, and healthcare providers. Further research is needed to explore the implications of this research.

**Acknowledgments**

Daisuke Matsubayashi and Masaki Nakamura from MDV supported data collection and analysis. Editorial assistance was provided by Write All, Inc.

**Disclosure**

This study was presented as an oral presentation at the *67th Annual Congress of Japan Clinical Ophthalmology,* October 31-November 3, 2013 in Yokohama, Japan and as a poster at the *ISPOR 16th Annual European Congress*, November 2-6, 2013 in Dublin, Ireland.

This study was supported by Bayer Yakuhin Ltd. Ms. Narimatsu and Drs. Adachi and Wang are employees of Bayer Yakuhin Ltd. At the time of the study, Sheng-Wen Huang was employed by Bayer Yakuhin Ltd and provided data analysis support.
